# Psychosocial judgements and perceptions of adolescents with acne vulgaris: A blinded, controlled comparison of adult and peer evaluations

**DOI:** 10.1186/1751-0759-5-11

**Published:** 2011-08-13

**Authors:** Eva Ritvo, James Q Del Rosso, Mark A Stillman, Christopher La Riche

**Affiliations:** 1 Department of Dermatology Miller School of Medicine, University of Miami, 4308 Alton Road, Suite 910, Miami Beach, FL, USA 33140; 2 Valley Hospital Medical Center, Las Vegas, NV, USA and Las Vegas Skin & Cancer Clinics, 880 Seven Hills Drive, Suite 260, Henderson, NV, USA 89052; 3 School of Liberal Arts, Georgia Gwinnett College, Lawrenceville, GA, USA 30043; 4 Department of Psychiatry and Behavioral Science, University of Miami Miller School of Medicine, 1695 NW 9th Ave Suite 3100, Miami, FL, USA 33136

## Abstract

**Background:**

The purpose of the current survey was to evaluate how teenagers and adults view teens with acne as compared to those with smooth, clear skin. We also surveyed teens and adults about their experiences with acne.

**Methods:**

We hypothesized that teens with acne would be perceived in a more negative fashion as compared to teens with smooth, clear skin. We presented digitally altered photographs to our responders and asked how they perceived the two groups. No mention was made of acne. In the first survey (n = 1,002), both adults and teens provided their impressions on photo images of teenagers with either clear skin or acne. In the second survey (n = 1,006), the adults and teens also answered questions about their own experiences with acne.

**Results:**

Survey 1. With respect to impressions of photo images, the first thing teens and adults noticed about a person with acne was their skin (65% and 75%, respectively). Teenagers with acne were perceived most often by other teens and adults (teen responder %, adult responder %) as being shy (39%, 43%), nerdy (31%, 21%), stressed (24%, 20%), lonely (23%, 22%), boring (15%, 6%), unkempt (13%, 7%), unhealthy (12%, 8%), introverted (9%, 23%), and rebellious (7%, 5%).

Survey 2. Most teenagers with acne (64%) felt embarrassed by it and thought that getting acne was the most difficult aspect of puberty (55%). Teenagers with acne reported lower self-confidence or shyness (71%); difficulty finding dates (43%), problems making friends (24%), challenges with school (21%), and trouble getting a job (7%).

**Conclusions:**

Teens with smooth, clear skin were rated higher on every favorable characteristic and lower on every unfavorable characteristic by both teens and adults. In most cases, the first thing that respondents noticed was the skin of teens with acne. Teenagers and adults alike perceived other teens with acne as generally being shy, less socially active, more likely to be bullied, and less successful in terms of finding a job. Overall, these results show that acne has a negative effect on the way people are perceived by others.

## Background

We all know that how you look affects how you feel. In their recent book, *The Beauty Prescription*, Luftman and Ritvo describe a beauty-brain loop in which inner beauty/outer beauty health and environment interact with one another. This survey took a unique look at how outer beauty impacts the way others view you [1].

Our ancestors recognized that being attractive was important for survival [1]. Beautiful skin, nails, hair and teeth were indicators of youth, good health and even reproductive capability. Attractiveness equated to good health and perpetuation of the species. This is one reason why men still find women in their late teens and early twenties so alluring. At this age, young women are most loudly broadcasting signals of their vitality and fertility through their faces and bodies. Conversely, any change from the ideal appearance (ie unhealthy skin, asymmetric facial or body features, being underweight or overweight) is perceived negatively.

Etcoff, in Survival of the Prettiest, stated, "Our extreme sensitivity to beauty is hard-wired, that is, governed by circuits in the brain shaped by natural selection [2]. We love to look at smooth skin, thick shiny hair, curved waists, and symmetrical bodies because in the course of evolution the people who noticed these signals and desired their possessors had more reproductive success."

The skin can be thought of as the most aesthetic organ of the body. One of the first things we notice about another person is their skin. On the average adult, skin weighs 8-10 lbs and covers about 22 square ft of surface area on the body [3]. Clear, healthy skin that is free of diseases such as acne is an important factor that contributes to our positive perceptions toward others.

Acne vulgaris (acne) is a common skin disease involving the pilosebaceous unit, including the follicular canal, hair follicle and sebaceous glands [4]. Microcomedones, caused by hyperproliferation and hyperkeratinization in the follicular canal, constitute the initial lesions in this disease [5]. The bacterial species *Propionibacterium acnes *colonizes the follicular channel. This stimulates cytokine production, which eventually leads to the formation of inflammatory lesions. Upon rupture of the follicular walls, granulomatous lesions and scarring often occurs [5]. The onset of acne is typically at adrenarche, which is one to two years before visible evidence of puberty (~age 10), however, acne vulgaris may start later, including in post-teenage years, especially in females [4,6].

The prevalence of acne is particularly high among teenagers and young adults. Ghodsi and colleagues found, in a study of 1,002 adolescents, that the prevalence of acne was 93.3% [7]. Fourteen (14%) percent of the participants suffered from moderate to severe acne. Collier et al. reported that 73.3% adults aged 20 years and older reported having acne at some point in their lives. The prevalence of acne was higher in women than it was in men [8]. Poli and colleagues found, in a study of 3,305 women aged 25-40, that the total acne prevalence was 41% [9]. Forty-nine (49%) percent of the respondents with acne had scars and/or pigmented macules.

The Global Alliance to Improve Outcomes in Acne Group recently published their update on the management of acne [6]. The group described acne as a chronic disease due to its prolonged course, pattern of recurrence, acute outbreaks, and slow onset. Due to the negative psychological and social effects of the disease, early and aggressive treatments were considered by the panel to be warranted.

The physical and emotional scars of acne can extend from adolescence and beyond. Many adolescents experience considerable psychological distress as a result of their acne [10]. Severe acne can cause extensive physical scarring, and can last well into the fourth and fifth decades [11]. This scarring can cause despair and other negative psychosocial effects. It is apparent that acne is a disease that causes many challenges to the psychological resources of a patient and these effects can last up to several decades.

Evidence abounds that teen acne leads to distress, higher rates of depression and in extreme cases treatment with isotretinoin has been associated with suicidal ideation and actions [12-15]. Antidepressants are used to combat the psychological symptoms of skin diseases such as acne [16]. Uhlenhake et al. reported that depression was two to three times more prevalent in acne patients than in the general population, with 8.8% of acne patients experiencing clinical depression [15]. Most cases of depression and antidepressant therapy utilization were observed in acne patients aged 18 and over.

Previously, it has been reported that people with acne suffer from a lack of confidence and self-esteem [17,18]. This survey attempted to assess whether acne changes how others perceive you.

## Methods

The Ritvo/AARS Perception Survey was conducted by Kelton Research between April 10th and April 24th, 2009 using an email invitation. Two separate national online surveys, one with a sample of 1,002 nationally representative adults age ≥ 18 and one with a sample of n = 1,006 teenagers aged 13-17. Both groups of participants took the same survey. Quotas were set to ensure reliable and accurate representation of each audience.

**Figure 1:**
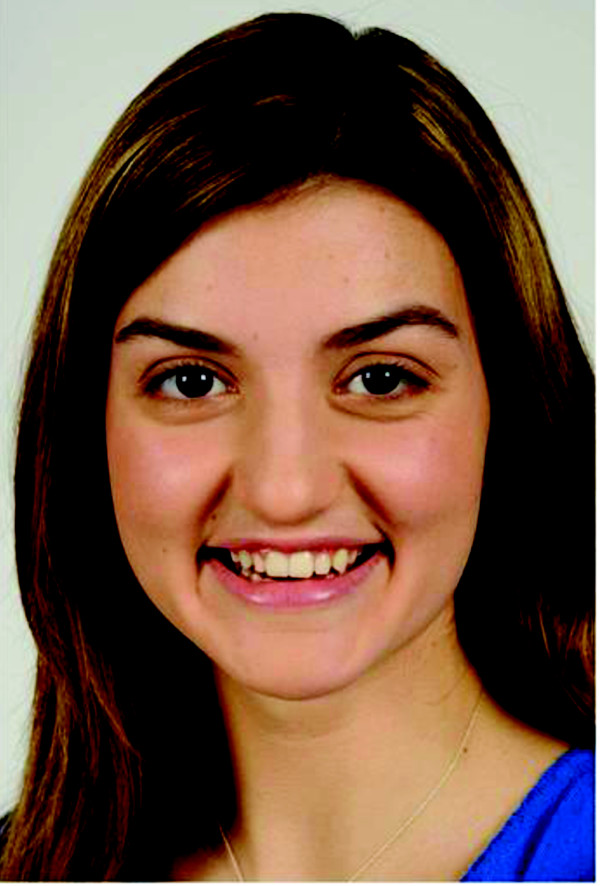
**Representative facial image of a teen with clear skin**.

**Figure 2:**
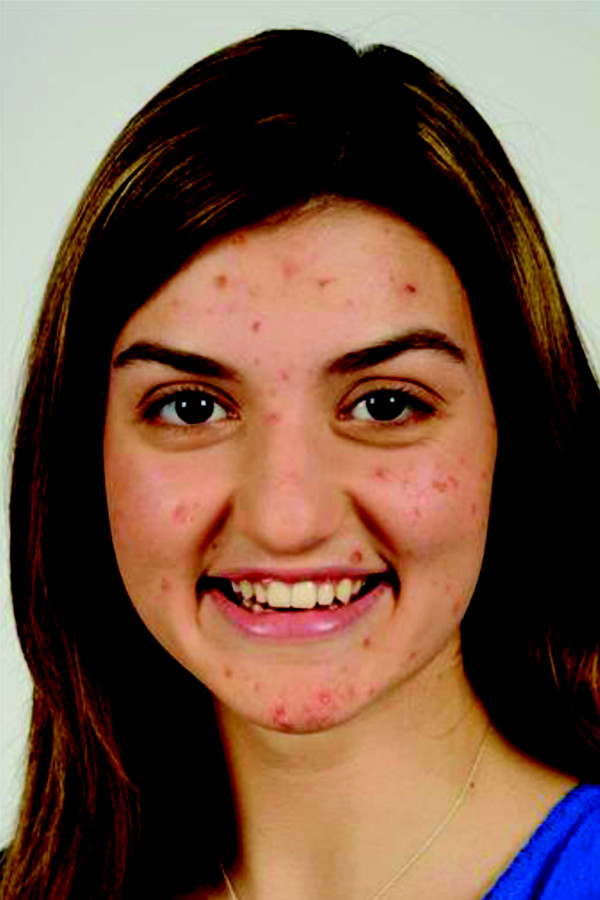
**Representative facial image of a teen with visible acne**.

Each respondent answered questions about several pictures of teens (males and females of Caucasian, African American, or Hispanic ethnicity) with or without acne (Figure 1 and 2). The total amount of visual stimuli used in the survey consisted of 12 pictures-based on a 2 × 3 × 2 exposure model (gender × race × acne/clear). Each respondent reacted to 3 randomly selected pictures, with the only condition being that it was a combination of either (a) 1 clear and 2 acne pictures or (b) 2 clear and 1 acne picture. This design allowed the respondents to focus in-depth on specific stimuli and kept the survey at a reasonable length. In an effort not to 'lead' the responses, acne was not specifically mentioned during the perception survey (Table 1). Respondents then answered questions about their own experiences with acne in the second survey (Table 2).

**Table 1:** Impressions from Teens (13-17 years of age) and Adults (≥ 18 years of age) after Looking at Photos of People with Either Clear Skin or Acne

	Teens		Adults	
	**Clear**	**Acne**	**Clear**	**Acne**

**Number of respondents (n)**	**1503**	**1508**	**1502**	**1504**

***Does this person look like any of your friends (or does this person remind your of your friends when you were a teenager)?***				

Yes	32%	23%	50%	47%

No	68%	77%	50%	53%

***Does this person resemble you (or resemble you when you were a teenager)?***				

Yes	7%	5%	23%	22%

No	93%	95%	77%	78%

***If you said yes, what is it that you have in common with this person?***				

Hair color	27%	19%	NA	NA

Skin or complexion	38%	59%	NA	NA

Eye color	16%	12%	NA	NA

Shape of face	12%	4%	NA	NA

Nose	4%	4%	NA	NA

Shape of mouth	4%	1%	NA	NA

***Just based on their face, how likely do you think it is that*** ***you'd be friends with this person?***				

Extremely likely	15%	8%	NA	NA

Somewhat likely	44%	36%	NA	NA

Likely net	59%	44%	NA	NA

Not likely or unlikely	21%	29%	NA	NA

Not very likely	12%	15%	NA	NA

Not at all likely	7%	12%	NA	NA

Not likely net	19%	27%	NA	NA

***If you were friends with this person, how likely would you be to post a picture of you and him or her on your Facebook or MySpace page?***				

Extremely likely	20%	9%	NA	NA

Somewhat likely	38%	32%	NA	NA

Likely net	58%	41%	NA	NA

Not likely or unlikely	19%	24%	NA	NA

Not very likely	12%	16%	NA	NA

Not at all likely	12%	19%	NA	NA

Not likely net	23%	35%	NA	NA

***Which of the following titles could this person potentially earn in a high school yearbook? Please choose all that apply***.				

Best personality	39%	34%	NA	NA

Most likely to succeed	33%	31%	NA	NA

Most spirited	30%	26%	NA	NA

Best dressed	20%	10%	NA	NA

Most artistic	25%	25%	NA	NA

Best looking	18%	5%	NA	NA

Class clown	19%	20%	NA	NA

Other	4%	5%	NA	NA

None of these	7%	14%	NA	NA

***How likely is it that this person is currently dating someone?***				

Extremely likely	16%	4%	20%	5%

Somewhat likely	44%	30%	47%	36%

Likely net	59%	34%	67%	41%

Not likely or unlikely	22%	29%	24%	36%

Not very likely	11%	21%	7%	18%

Not at all likely	8%	16%	2%	5%

Not likely net	19%	37%	9%	23%

***If you had to choose one, is this person more likely to be a leader or a follower?***				

A leader	49%	29%	54%	34%

A follower	51%	71%	46%	66%

***If you had to decide, is this person likely to be bullied by someone?***				

Yes	33%	60%	29%	56%

No	67%	40%	71%	44%

***How many close friends do you think this person has? Your best guess is fine***.				

None	2%	3%	1%	1%

1	5%	10%	5%	9%

2	10%	18%	10%	19%

3-4	23%	26%	24%	27%

5-6	23%	19%	28%	26%

7-9	8%	6%	7%	4%

10-14	11%	8%	15%	9%

15 or more	18%	10%	11%	6%

***If you had to choose one, which of the following do you believe this person would do for fun on a typical weekend? Please choose one answer in each question***.				

*Go on a date or stay at home with parents*				

Go on a date	60%	36%	64%	42%

Stay at home with parents	40%	64%	36%	58%

*Go out with a large group of friends or study*				

Go out with a large group of friends	60%	42%	57%	44%

Study	40%	58%	43%	56%

*Play group sports such as basketball or play videogames alone*				

Play group sports such as basketball	66%	47%	69%	50%

Play videogames alone	34%	53%	31%	50%

Read a book or go to the movies				

Go to the movies	64%	46%	65%	51%

Read a book	36%	54%	65%	49%

***Which of the following extra-curricular activities is this person*** ***likely to participate in? Please choose all that apply***.				

Chorus or other singing club	33%	31%	41%	33%

Student government	41%	41%	53%	44%

Drama club or school play	40%	33%	46%	33%

School subjects clubs or study groups, such as math, history, or foreign languages	36%	41%	45%	50%

Debate team	36%	41%	36%	33%

Cheerleading	21%	9%	22%	8%

***Organized sports, such as football, tennis, field hockey, gymnastics, basketball, or track***				

Marching band	28%	39%	31%	40%

Computer or other technical club	26%	35%	37%	44%

Chess club	21%	36%	18%	26%

Martial arts	16%	15%	13%	10%

Other	2%	2%	1%	1%

None of these	2%	5%	3%	4%

***What do you think this person's grade point average is? Your best guess is fine. Please assume a 100-point scale***.				

Less than 50%	10%	10%	5%	5%

50-59%	2%	3%	1%	1%

60-69%	2%	2%	1%	2%

70-79%	8%	8%	8%	10%

80-89%	28%	26%	34%	31%

90-99%	40%	41%	44%	45%

100%	10%	11%	7%	6%

***If this person were to attend college, which of the following best describes the type of college he or she would attend?***				

State college	38%	34%	48%	44%

Ivy League (Harvard, Yale, MIT, etc.)	24%	26%	16%	13%

Small private college	14%	16%	15%	13%

Community college	17%	17%	17%	23%

Trade school	6%	6%	3%	5%

Other	1%	1%	1%	1%

***How strongly do you agree with the following statement: This person is a good representation of the average American teenager***.				

Strongly agree	17%	14%	22%	18%

Somewhat agree	45%	41%	57%	56%

Agree net	63%	56%	78%	74%

Neither agree nor disagree	10%	10%	5%	5%

Somewhat disagree	21%	23%	13%	17%

Strongly disagree	7%	11%	3%	4%

Disagree net	28%	34%	16%	21%

How likely is this person to be professionally successful?				

Extremely likely	29%	24%	30%	20%

Somewhat likely	50%	49%	54%	57%

Likely net	78%	73%	84%	78%

Not likely or unlikely	17%	20%	14%	19%

Not very likely	3%	4%	1%	3%

Not at all likely	2%	3%	0%	1%

Not likely net	5%	7%	2%	4%

How likely would you be to hire this person ***for an after-school or summer job?***				

Extremely likely	NA	NA	28%	13%

Somewhat likely	NA	NA	52%	49%

Likely net	NA	NA	79%	62%

Not likely or unlikely	NA	NA	18%	28%

Not very likely	NA	NA	2%	7%

Not at all likely	NA	NA	1%	2%

Not likely net	NA	NA	3%	10%

***How likely would you be to hire this person as a babysitter?***				

Extremely likely	NA	NA	19%	11%

Somewhat likely	NA	NA	35%	33%

Likely net	NA	NA	54%	44%

Not likely or unlikely	NA	NA	29%	31%

Not very likely	NA	NA	11%	15%

Not at all likely	NA	NA	6%	9%

Not likely net	NA	NA	17%	25%

Questions are in italics. NA, not applicable, this question was not asked of this study group of participants.

**Table 2:** Responses of Teenagers and Adults on Their Experiences with Acne

*How important to you is the way you look? (For respondents over age 18 with teen children: How important is personal appearance to your teen?)*				
	**Teens**		**Adults**	

**Response**	**n**	**%**	**n**	**%**

Extremely important	415	41%	90	43%

Somewhat important	509	51%	101	49%

Important net	924	92%	191	92%

Not very important	67	7%	16	8%

Not at all important	15	1%	1	< 1%

Not important net	82	8%	17	8%

***How important is the way you look to your parent(s) or guardian(s)? (For respondents over age 18 with teen children: How important is your teen's personal appearance to you?)***				

Extremely important	249	25%	57	27%

Somewhat important	377	37%	130	63%

**Important net**	626	62%	187	90%

Not very important	271	27%	20	10%

Not at all important	109	11%	1	< 1

**Not important net**	380	38%	21	10%

***What's the most difficult aspect of puberty?***				

Getting acne	554	55%	451	45%

Your body changing	217	22%	403	40%

Body odor	125	12%	41	4%

Your voice changing	37	4%	24	2%

Other	73	7%	83	8%

***Which of the following do you think is the most difficult to have in high school?***				

Extra weight	568	56%	529	53%

Acne	339	34%	407	41%

Braces	67	7%	41	4%

Glasses	32	3%	25	2%

***Have you ever had acne?***				

Yes, and I still Have it	543	54%	NA	NA

Yes, but I don't currently have it	282	28%	NA	NA

No	181	18%	NA	NA

***Which of the following best describes your acne most of the time? (Among respondents who have ever had acne). (For respondents over 18 with teen children: Which of the following best describes your teen's acne most of the time?)***				

Severe: Numerous red and inflamed pimples cover large parts of my face or other parts of my body	59	7%	9	9%

Moderate: Pimples are frequent, inflamed, and red	336	41%	55	52%

Mild: Occasional pimples that clear up on their own	430	52%	41	39%

***If you have ever had acne, which of the following best describes you?***				

My acne existed only as a teenager	NA	NA	310	31%

My acne lasted into adulthood before disappearing	NA	NA	131	13%

My acne lasted into adulthood, and I still have it	NA	NA	202	20%

I have never had acne	NA	NA	359	36%

***Did your acne bother you more as a teen, or as an adult? (Among respondents who had acne both as teens and adults)***				

Teen	NA	NA	212	64%

Adult	NA	NA	121	36%

***Have you ever been embarrassed by your acne? (Among respondents who have ever had acne)(For respondents over 18 with teen children: Have you ever been embarrassed by your teen's acne?)***				

Yes	527	64%	18	17%

No	298	36%	87	83%

***Do your parents or other adults in your life have acne?***				

Yes	378	38%	NA	NA

No	628	62%	NA	NA

***About what percentage of your friends have acne? Your best guess is fine***.				

**Response**	**n**	**%**	**n**	**%**

None	50	5%	NA	NA

1-9%	173	17%	NA	NA

10-19%	111	11%	NA	NA

20-29%	119	12%	NA	NA

30-49%	133	13%	NA	NA

50-69%	203	20%	NA	NA

70-99%	195	19%	NA	NA

100%	22	2%	NA	NA

***Which of the following would you be willing to do if you could get rid of your acne forever? Please choose all that apply. (Among respondents who have ever had acne)***				

Stay off Facebook for a year	485	59%	NA	NA

Not go on a date for a year	247	30%	NA	NA

Take my mom or dad as a date to my prom	109	13%	NA	NA

Have my grade point average drop a lot	91	11%	NA	NA

Other	123	15%	NA	NA

***Which of the following, if any, have you ever done to address your teen's acne? (Among respondents whose teen children have acne)***				

Encouraged a proper skincare regimen such as washing their face twice a day	NA	NA	78	74%

Purchased medication over the counter	NA	NA	74	70%

Taken them to a doctor for guidance or a prescription treatment	NA	NA	43	41%

Made changes in their diet such as avoiding chocolate or caffeine	NA	NA	29	28%

Other	NA	NA	3	3%

I haven't done anything to address my teen's acne	NA	NA	6	6%

***Which of the following, if any, did you do to address your acne as a teenager? Please choose all that apply. (Among respondents who had acne as teens and adults)***				

Purchased medication over the counter	NA	NA	352	55%

Started a proper skincare regimen such as washing my face twice a day	NA	NA	345	54%

Visited a doctor for guidance or a prescription treatment	NA	NA	148	23%

Made changes in my diet such as avoiding chocolate or caffeine	NA	NA	144	22%

Other	NA	NA	19	3%

I didn't do anything to address my acne as a teenager	NA	NA	98	15%

***Which of the following have you ever done to deal with your acne?*** ***(Among respondents who have ever had acne)***				

Popped or picked it	638	77%	485	75%

Applied over-the-counter acne medication	481	58%	466	72%

Applied hot water or steam to your face	398	48%	269	42%

Applied rubbing alcohol	280	34%	232	36%

Used a prescription medication	274	33%	173	27%

Applied another substance not intended for acne, such as baking soda or toothpaste	225	27%	142	22%

Other	40	5%	23	4%

I have never done anything to deal with my acne	28	3%	31	5%

***Which of the following have you ever done to hide your acne?*** ***(Among respondents who have ever had acne)***				

Used concealer or other makeup to cover it	398	48%	336	52%

Tried to comb or style hair over it	343	42%	136	21%

Refused to get your picture taken	192	23%	119	19%

Avoided social situations, such as a party	168	20%	138	21%

Covered it with clothing, such as a scarf or hat	104	13%	38	6%

Worn a band-aid over it	66	8%	67	10%

Cancelled a date	52	6%	34	5%

Other	13	2%	5	1%

None of these	198	24%	201	31%

***Have you ever sought advice from a doctor for your acne?*** ***(Among respondents who have sought medical advice for their acne)***				

Yes	261	32%	203	32%

No	564	68%	440	68%

***How many different types of prescription acne treatment have you tried? Please think of treatments such as creams, pills, gels, and washes***. ***(Among respondents who have sought medical advice for their acne)***				

None	8	3%	9	4%

1	25	10%	37	18%

2	51	20%	51	25%

3-4	76	29%	48	24%

5-9	56	21%	31	16%

10 or more	45	17%	27	13%

***Which of the following best describes the majority of prescription acne treatments you've tried? (Among respondents who have sought medical advice for their acne and tried treatment)***				

They completely cleared my skin	17	7%	24	12%

A major improvement, but my skin wasn't completely clear	57	23%	35	18%

A noticeable improvement, but I still had some acne	74	29%	59	30%

A little improvement, but not much	84	33%	59	30%

No improvement	21	8%	17	9%

***How effective was the best prescription acne treatment you've ever tried? (Among respondents who have sought medical advice for their acne and tried treatment)***				

Extremely effective	70	28%	46	24%

Somewhat effective	142	56%	111	57%

**Effective Net**	212	84%	157	81%

Not very effective	31	12%	33	17%

Not at all effective	10	4%	4	2%

**Not effective net**	41	16%	37	19%

***Which of the following reasons have you ever had for not treating your acne or putting off treatment of your acne? Please choose all that apply. (Among respondents who have ever had acne)***				

My acne is or was not severe enough to require treatment	340	41%	327	51%

Acne treatments I've used in the past haven't worked	277	34%	123	19%

It's too expensive	206	25%	201	31%

Acne treatments are too expensive	196	24%	166	26%

I've heard acne treatments do not work	177	21%	59	9%

I'm afraid of possible side effects	156	19%	73	11%

Other	37	4%	20	3%

None of these	111	13%	94	15%

Abbreviation: NA, not applicable, this question was not asked of this study group of participants. Survey questions are in italics.

Results of any sample are subject to sampling variation. The magnitude of the variation is measurable and is affected by the number of responses and the level of the percentages expressing the results. For this survey, the overall margin of error for each group (teenagers and adults) was ± 3.1 percentage points at the 95% confidence level.

## Results

### Respondents Impressions of Photos of Clear Skin Compared to Skin with Acne-(Table 1)

**Figure 3:**
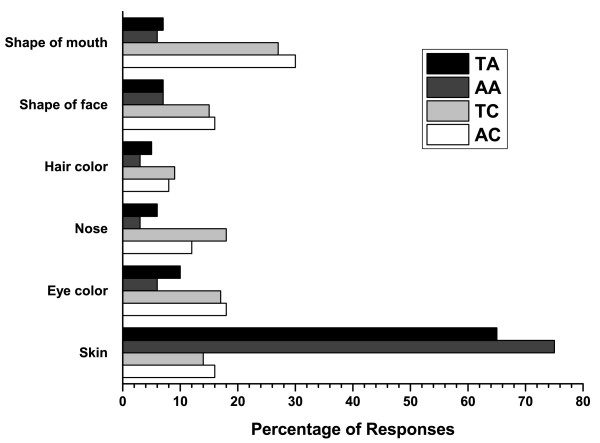
**Responses to the question: If you had to choose one, what's the first thing you noticed about this person's face?**. TA = Teens viewing photos of teens with acne, AA = Adults viewing photos of teens with acne, TC = Teens viewing photos of teens with clear skin, AC = Adults viewing photos of teens with clear skin.

**Figure 4:**
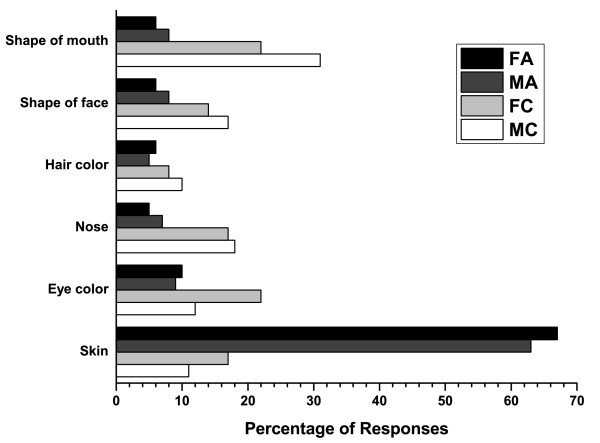
**Responses by gender from teenagers: If you had to choose one, what's the first thing you noticed about this person's face?**. FA = Female teens viewing photos of teens with acne, MA = Male teens viewing photos of teens with acne, FC = Female teens viewing photos of teens with clear skin, MC = Male teens viewing photos of teens with clear skin.

**Figure 5:**
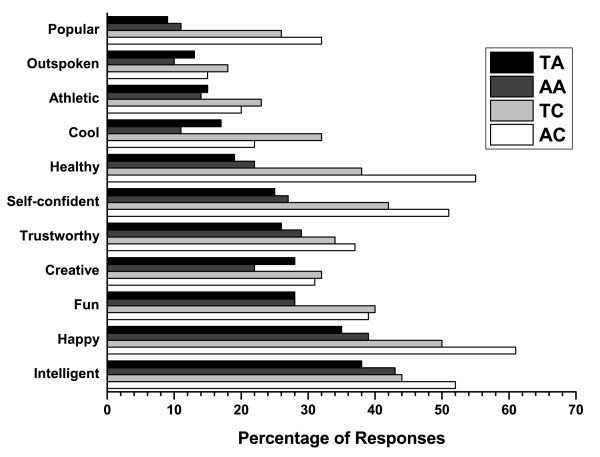
**Teenagers' and adults' perceptions when asked: Which of the following words or traits describe this person?**. Positive traits. TA = Teens viewing photos of teens with acne, AA = Adults viewing photos of teens with acne, TC = Teens viewing photos of teens with clear skin, AC = Adults viewing photos of teens with clear skin.

**Figure 6:**
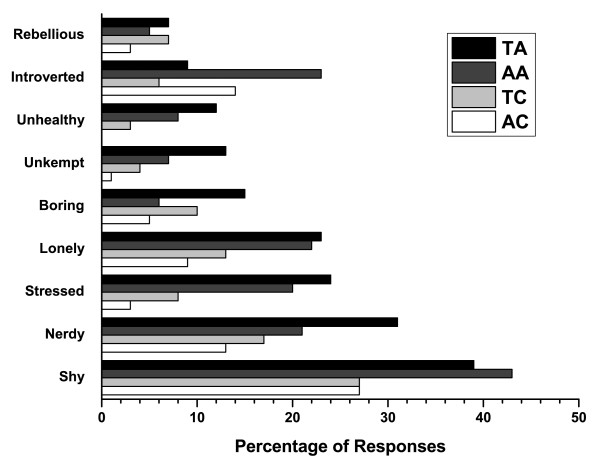
**Teenagers' and adults' perceptions when asked: Which of the following words or traits describe this person?**. Negative traits. TA = Teens viewing photos of teens with acne, AA = Adults viewing photos of teens with acne, TC = Teens viewing photos of teens with clear skin, AC = Adults viewing photos of teens with clear skin.

Representative photos of a teen with and without acne are shown in Figures 1 and 2. Overall, most teenagers (65%; 75% for adults) noticed the skin first for the photos of a person with acne compared with only 14% (16% for adults) for the photos of a person with clear skin (Figure 3). When the results were analyzed by gender, the skin was the first thing that both female and male teens noticed from photos the most (Figure 4). Teenagers with clear skin (teen responder %, adult responder %,) were thought of as being happy (50%, 61%), healthy (38%, 55%), intelligent (44%, 52%), self-confident (42%, 51%), fun (40%, 39%), trustworthy (34%, 37%), creative (32%, 32%), popular (26%, 32%), cool (32%, 22%), athletic (23%, 20%), and outspoken (18%, 15%) compared with their colleagues with acne (Figure 5). Teens with acne were perceived most often by adults and other teens as being shy (39%, 43%), introverted (9%, 23%), lonely (23%, 22%), nerdy (31%, 21%), stressed (24%, 20%), unhealthy (12%, 8%), unkempt (13%, 7%), boring (15%, 6%), and rebellious (7%, 5%) compared to their counterparts with clear skin (Figure 6).

**Figure 7:**
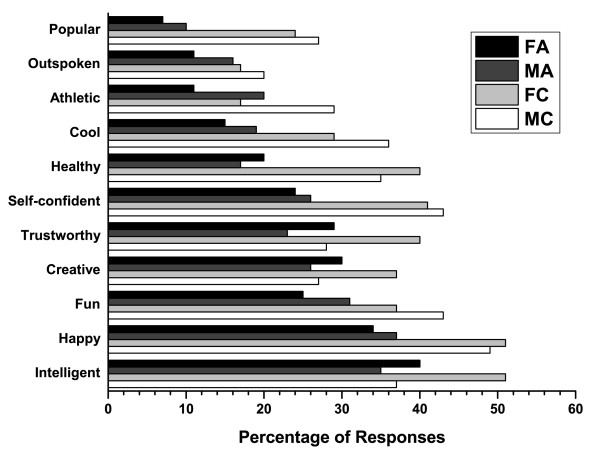
**Responses by gender from teenagers: Which of the following words or traits describe this person?**. Positive traits. FA = Female teens viewing photos of teens with acne, MA = Male teens viewing photos of teens with acne, FC = Female teens viewing photos of teens with clear skin, MC = Male teens viewing photos of teens with clear skin.

**Figure 8:**
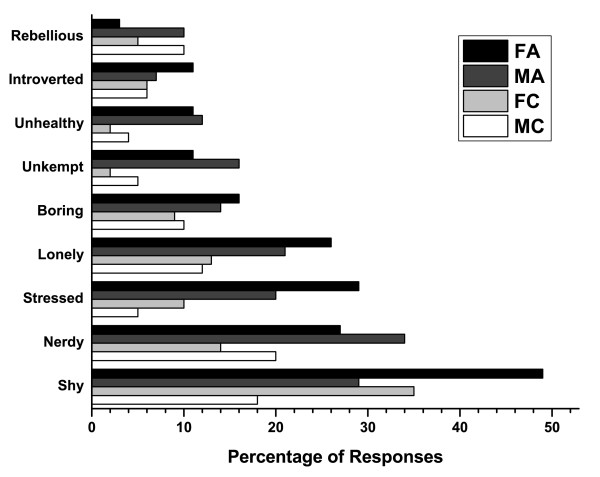
**Responses by gender from teenagers: Which of the following words or traits describe this person?**. Negative traits. FA = Female teens viewing photos of teens with acne, MA = Male teens viewing photos of teens with acne, FC = Female teens viewing photos of teens with clear skin, MC = Male teens viewing photos of teens with clear skin.

When the perception data from teenagers was subdivided by gender, a higher proportion of female teens with clear skin were perceived as being intelligent (51%), happy (51%), trustworthy (40%), healthy (40%), and creative (37%), by more survey respondents than females with acne or males with or without acne (Figure 7). A higher percentage of male teenagers with clear skin were thought to be self-confident (43%), fun (43%), cool (36%), athletic (29%), popular (27%), and outspoken (20%) than other teens. Conversely, girls with acne were perceived as being shy (49%), stressed (29%), lonely (26%), boring (16%), and introverted (11%) by more survey respondents than their colleagues (Figure 8). More boys with acne were perceived as being nerdy (34%), unkempt (16%), and unhealthy (12%) than their counterparts. Based on the facial images, 59% of respondents said they would likely be friends with the person with clear skin versus 44% for the person with visible acne (Table 1).

### Survey results regarding the respondents own experiences with acne (Table 2)

**Figure 9:**
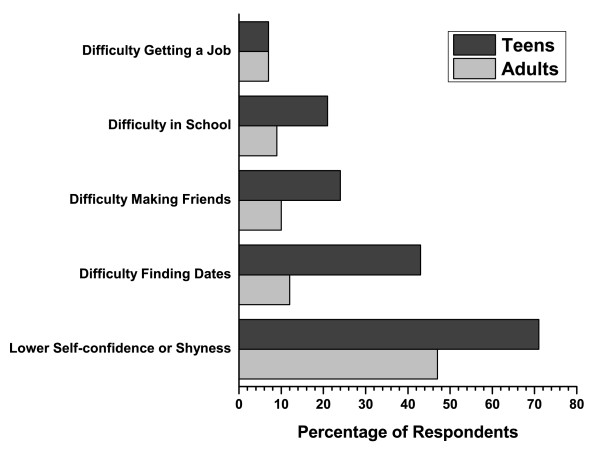
**Responses to the survey question: Which of the following are effect of having acne?**. Not shown in the graph above; None of these = 14%, Other = 4%.

**Figure 10:**
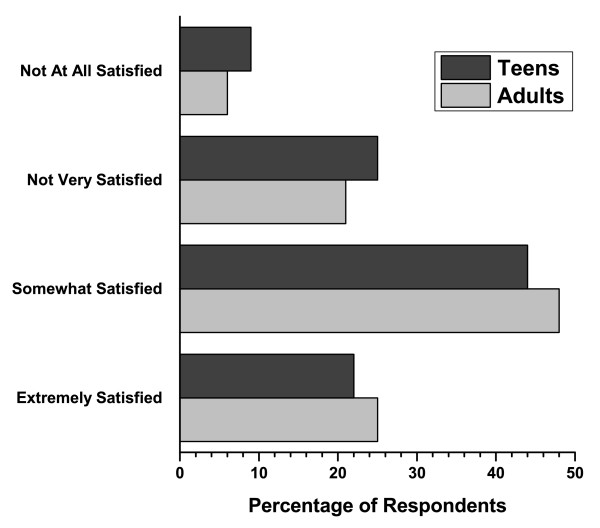
**Responses to the survey question: How satisfied were you with the advice or treatment/medication you received form your doctor for your acne?**.

The majority of respondents in both age groups (92%) thought that the way they look is important. Fifty-five percent (55%) of teenagers 13-17 and 45% of respondents aged ≥ 18 felt that getting acne was the most difficult aspect of puberty. Most teenagers with acne (64%) were embarrassed by it, while only 17% of parents found their teenager's acne a source of embarrassment. Teenagers with acne reported lower self-confidence or shyness (71%), difficulty finding dates (43%), problems making friends (24%), challenges with school (21%), and trouble getting a job (7%)(Figure 9). Most teenagers (68%) had not sought medical advice for their acne. Most of the teen respondents who had ever had acne indicated that they would stay off Facebook for a year (59%) or not go on a date for a year (30%) if they could get rid of their acne forever. Approximately 67% of teens ages 13-17 have tried 3 or more prescription acne treatments with 84% reporting that these treatments were effective. Most teens (66%) and adults (73%) were satisfied with the advice or treatment/medication they received from their doctors for their acne (Figure 10).

## Discussion

Survey results clearly show and confirmed the hypothesis that acne affected how people were perceived. Both the adults and teenagers were more likely to first notice the skin of teens with acne. In contrast, when shown images of teens with clear skin, a variety of physical features were noticed first (ie shape of mouth, eye color, nose). Teenagers with clear skin were more commonly described as being happy, intelligent, self-confident, healthy, and fun. Interestingly, those with acne were most often perceived as being shy, stressed, unkempt, lonely, boring, nerdy, and introverted. Therefore, one might imply from these results that how you look affects how others view you.

Greater acne severity has been shown to be associated with poorer social outcomes and quality of life [19]. Dermatological-related social anxiety appears to be negatively associated with intention to participate in sport/exercise, self-esteem and dermatological quality of life [18]. Among seven facial disfigurements, acne had the largest negative impact on finding a partner [20]. In the current survey people with acne were thought less likely to be currently dating someone. Murray and Rhodes found that 5 themes were revealed in interviews with acne patients: powerlessness and the variable nature of acne; comparisons, self-image and identity; the experience of general social interaction; relationships with family and friends; and gender, sexuality, and romantic relationships [21].

Many of the negative psychological effects on those individuals who are afflicted by acne relate to a diminished perception of attractiveness [22]. Acne has been termed by some investigators to be a biopsycosocial skin condition [19]. Motley and Finlay reported that patients with facial acne felt that they were unattractive, reluctant to engage in dating activities, and were socially isolated [23]. There was a sense among adults and teenagers in the current survey that the lives of teens with acne are less fulfilling, with more weekend time spent alone. Adolescents with acne have reported lower scores for total body esteem, sexual attractiveness, and weight concerns than their clear-skinned colleagues [17].

Rubinow and colleagues conducted a survey of 55 acne patients to evaluate psychiatric morbidity and mood characteristics [14]. The most frequently reported adverse psychological effects of a severe form of acne called cystic acne included embarrassment, self-consciousness, social isolation, decreased physical activity, anxiety with the opposite sex, decreased self-confidence, preoccupation with acne, sleep disturbances, nuisance, feeling ill at ease, frustration, and decreased self-esteem. In our survey, a higher percentage of teens with clear skin were perceived as being self-confident. Nearly two in three (64%) teenagers acknowledged that acne has been a source of embarrassment. Magin and colleagues also found skin diseases like acne to be a cause for embarrassment and impaired self-esteem [24].

Not surprisingly, most (69%) teenagers with acne wished their physical appearance was better in some way, compared to 50% of those who have either never had it or don't have it currently. Acne caused so must frustration that nearly three in five teenagers would stay off Facebook for an entire year if they could get rid of their acne forever. Interestingly, 13% would actually pick one of their parents as a prom date if they could eliminate their acne for the rest of their lives. These responses underscore the value that teenagers place on their appearance and the lengths they would go to treat their acne.

Our survey suggests that acne can have a negative effect on relationships and social life. A smaller percentage of teenagers admitted that they would be friends with someone who had acne. More than 3-fold more teenagers than parents believe acne can cause difficulty for teens in regards to finding dates. Yet both adults and teenagers concur that in an average weekend, the teens with acne would be more likely than clear-skinned teens to stay at home with their parents than go out on a date. Overall, fewer teenagers with acne are described as likely to be in a romantic relationship by both adults and their teenage peers. A teen's social life can be negatively affected by acne-one in five have avoided social situations to keep blemishes a secret, and some have even cancelled a date.

Gender differences relating to the social pressures around acne and appearance have been reported in the literature. Girls were found to have higher levels of emotional and social impairments due to their acne [25]. Brook et al. studied the extent to which acne influences the emotional life of adolescent girls [26]. Neuroticism (anxiety level) was the first predictor of the sense of coherence measure for girls suffering from acne, followed by extraversion and psychoticism. One study showed that female students with acne were significantly more depressed than male students [27]. In our survey, more female teens with clear skin were perceived as being intelligent, happy, trustworthy, creative, and healthy compared with their colleagues. Conversely, a higher percentage of girls with acne were perceived as being shy, stressed, lonely, boring, and introverted. Teenaged boys with clear skin were thought by a higher proportion of respondents to be self-confident, fun, cool, athletic, popular, and outspoken than other teens. More male teens with acne were perceived as being more nerdy, unkempt, and unhealthy than their counterparts. However, regardless of gender, the first thing that both males and females noticed in photos of teens with acne was their skin.

Social bullying that occurs at school is a problem that has recently gained a great deal of media attention. Most respondents from the current survey perceived that teens with acne are likely to be bullied, compared to a third who felt this about images of people with clear skin. Taunting, teasing, and bullying can be real problems for individuals with acne [24]. Teasing can be used as a means of social exclusion and establishing or maintaining dominance. Furthermore, this taunting behavior can perpetuate the lack of self-esteem and resultant depression in teenagers with acne.

Even with the extensive literature regarding acne's effects on behaviors, it is difficult to measure the short- and long-term psychosocial effect of this skin disorder [28]. Social interactions have changed with the times, in particular with the practically unlimited access to social networking sites on the Internet. Our modern preoccupation with blemish-free skin has increased the fear and emotional distress associated with acne. Adolescents, in particular, are an emotionally vulnerable population, and acne can place a significant burden on their psychological resources [29]. Over half of the teenagers in the current survey complained that acne is the most difficult aspect of puberty, even worse than their bodies changing, body odor, or changes in their voice. A higher percentage of adults and teenagers felt that acne causes more difficulty than other sources of embarrassment like braces or glasses.

The deleterious psychosocial effects of acne can be correlated with a reduction in employment opportunities. In studies by Mojon-Azzi et al., acne had the greatest negative impact on finding employment of all the facial anomalies tested [30,31]. Employment experience reports have shown limited opportunities resulting from acne and other skin diseases [28]. This may be due to the overall negative perceptions-being shy, nerdy, stressed and lonely-that tend to be linked to acne. In our survey, adults were less likely to consider hiring teenagers with blemished skin than the teens with clear skin. It has been shown that early employment and good work habits tend to translate into long term employment success [32].

Unfortunately, there may be a lack of understanding regarding the importance of seeking medical advice for the treatment of acne. Less than half of the parents in our survey have taken the initiative to take their teenagers to doctors for any kind of assessment, guidance or treatment for their acne. Many individuals do not go beyond basic efforts to deal with outbreaks of acne. Less than a third of teenagers and adults have taken the initiative to seek a doctor's advice about their own acne. Only about one third of respondents that ever had acne used a prescription medication to deal with their acne and the most popular reason for avoiding treatment was the simple belief that their skin condition wasn't bad enough to demand medical attention. However, 67% of teenagers 13-17 (who have sought medical advice for their acne) have tried 3 or more prescription acne treatments with about 84% reporting that these treatments were effective. Most teens and adults were satisfied with the advice or treatment/medication they received from their doctors for their acne.

The current survey had some limitations due to its survey nature. The participants were limited to people who were willing and able to respond to an online survey.

Future perception assessments may involve expanding the survey to other regions outside the US to see if cultural differences play a role in perception and whether they differ from the US responses. In addition, it would be interesting to evaluate perceptions of acne from different ethnic groups. Peoples' perceptions regarding visible acne scarring could be another area of future research.

## Conclusions

In most cases, the first thing that respondents noticed was the skin of teens with acne. Teenagers and adults alike perceived other teens with acne as generally being shy, less socially active, more likely to be bullied, and less successful in terms of finding a job. The analysis by gender showed that a higher percentage of girls with acne were perceived as being shy, stressed, lonely, boring, and introverted while a higher proportion of their male counterparts with acne were perceived as being nerdy, unkempt, and unhealthy compared with their colleagues. In addition, most teens have been embarrassed by their acne and would be willing to make a substantial sacrifice if they could remain free of acne for the rest of their lives. Teenagers with acne reported lower self-confidence or shyness, difficulty finding dates, problems making friends, challenges with school, and trouble getting a job. Most teens who have sought medical advice for their acne have tried 3 or more prescription acne treatments and reported that these treatments were effective. Overall, these results show that acne has a negative effect on the way people are perceived by the others.

## Competing interests

An educational grant to Dr. Ritvo was provided by Galderma Laboratories, L.P. Dr. Del Rosso has served as a consultant, researcher and/or speaker for several companies that have acne products in their company portfolio: Allergan, Coria, Galderma, Intendis, Medicis, Obagi Medical Products, Onset Therapeutics, Ortho Dermatology, Pharmaderm, Ranbaxy, Stiefel, TriaBeauty, Triax, Warner-Chilcott. Drs. Stillman and La Riche have no competing interests to disclose.

## Authors' contributions

ER-Conception, design, and interpretation of the study; writing and revising the article; final approval. JQDR-Data interpretation; revising the article; final approval. MAS-Conception and design of the study; revising the article; final approval. CLR-Conception and design of the study; revising the article; final approval.
